# Sustained Use of Patient Portal Features and Improvements in Diabetes Physiological Measures

**DOI:** 10.2196/jmir.5663

**Published:** 2016-07-01

**Authors:** Stephanie L Shimada, Jeroan J Allison, Amy K Rosen, Hua Feng, Thomas K Houston

**Affiliations:** ^1^Center for Healthcare Organization and Implementation Research (CHOIR)Edith Nourse Rogers Memorial Veterans HospitalBedford, MAUnited States; ^2^Department of Health Law, Policy, and ManagementBoston University School of Public HealthBoston, MAUnited States; ^3^Division of Health Informatics and Implementation ScienceDepartment of Quantitative Health SciencesUniversity of Massachusetts Medical SchoolWorcester, MAUnited States; ^4^Center for Healthcare Organization and Implementation Research (CHOIR)VA Boston Healthcare SystemBoston, MAUnited States; ^5^Department of SurgeryBoston University School of MedicineBoston, MAUnited States

**Keywords:** personal health records, diabetes mellitus, type 2, self care, HbA1c, cholesterol, LDL, blood pressure, veterans

## Abstract

**Background:**

Personal health records (PHRs) have the potential to improve patient self-management for chronic conditions such as diabetes. However, evidence is mixed as to whether there is an association between PHR use and improved health outcomes.

**Objective:**

The aim of this study was to evaluate the association between sustained use of specific patient portal features (Web-based prescription refill and secure messaging—SM) and physiological measures important for the management of type 2 diabetes.

**Methods:**

Using a retrospective cohort design, including Veterans with diabetes registered for the My Health *e* Vet patient portal who had not yet used the Web-based refill or SM features and who had at least one physiological measure (HbA1c, low-density lipoprotein (LDL) cholesterol, blood pressure) in 2009-2010 (baseline) that was above guideline recommendations (N=111,686), we assessed portal use between 2010 and 2014. We calculated the odds of achieving control of each measure by 2013 to 2014 (follow-up) by years of using each portal feature, adjusting for demographic and clinical characteristics associated with portal use.

**Results:**

By 2013 to 2014, 34.13% (38,113/111,686) of the cohort was using Web-based refills, and 15.75% (17,592/111,686) of the cohort was using SM. Users were slightly younger (*P*<.001), less likely to be eligible for free care based on economic means (*P*<.001), and more likely to be women (*P*<.001). In models adjusting for both features, patients with uncontrolled HbA1c at baseline who used SM were significantly more likely than nonusers to achieve glycemic control by follow-up if they used SM for 2 years (odds ratio—OR=1.24, CI: 1.14-1.34) or 3 or more years (OR=1.28, CI: 1.12-1.45). However, there was no significant association between Web-based refill use and glycemic control. Those with uncontrolled blood pressure at baseline who used Web-based refills were significantly more likely than nonusers to achieve control at follow-up with 2 (OR=1.07, CI: 1.01-1.13) or 3 (OR=1.08, CI: 1.02-1.14) more years of Web-based refill use. Both features were significantly associated with improvements in LDL cholesterol levels at follow-up.

**Conclusions:**

Although rates of use of the refill function were higher within the population, sustained SM use had a greater impact on HbA1c. Evaluations of patient portals should consider that individual components may have differential effects on health improvements.

## Introduction

Diabetes affects over 29 million Americans [1] and was estimated to have cost between $245 billion [2] and $322 billion [3] in 2012. Despite advances in effective treatments [4], almost half of those with type 2 diabetes do not meet recommended targets for glycemic control, low-density lipoprotein (LDL) cholesterol control, or blood pressure control [5]. Poor control of diabetes is associated with poor health outcomes, increased morbidity, and mortality [1,3]. Type 2 diabetes affects a large portion of US Veterans, with 25% of Veterans having the diagnosis [6,7].

Patients with diabetes and other chronic diseases do not do well with episodic, transactional care limited to in-person visits. The Institute of Medicine [8] has called for a shift toward continuous, coordinated care, leveraging information technology to support self-management and communication between clinic visits. Type 2 diabetes requires patient self-management and effective patient–provider communication to tailor treatments, manage side effects, monitor physiological processes, and screen for complications. Personal health records (PHRs) and patient portals are technologies with the potential to increase patient self-management and enable patients to better communicate with their clinical teams [9,10].

Evidence for patient portal effectiveness for chronic disease management is limited, and association with outcomes is mixed [11]. Tenforde et al [12] found that portal use was associated with improvements in diabetes-related quality measures but did not find a dose-response association with varying intensity of portal use and did not separate out effects by specific portal feature. Potential benefits of portal use have included patient reports of enhanced satisfaction, improved access outside of face-to-face visits, and improved efficiency and quality of face-to-face visits [13]. Studies from Kaiser [14] and Group Health Cooperative [15,16] found significant associations between use of secure messaging (SM) and improvements in diabetes care, with significant performance improvements in glycemic testing and control. Other studies have documented improvements in medication adherence among diabetic patients on statins exclusively using Web-based prescription refill through a patient portal [17]. Association of portal use and improvements in cholesterol and blood pressure effectiveness of care measures [14] have also been documented among patients with diabetes and hypertension.

Portals vary widely, adding to the difficulty in evaluating any effects they may have on patients’ health outcomes. Some are tethered to a health care system, others are not, some are disease specific, whereas most are not [18,19]. The Department of Veterans’ Affairs provides its patients with a portal, My HealtheVet (MHV), including features allowing them to refill VA prescriptions and send secure messages to their providers [20-22]. These two features, SM and Web-based prescription refill, are among the most common across portals and are the most frequently used [23]. Veterans with diabetes have relatively high adoption of MHV and of these key features [7].

This study examines whether diabetes outcomes are improved for patients with type 2 diabetes who initiate use of key features of the MHV patient portal compared with similar patients with type 2 diabetes who are also registered for the portal but do not initiate use of any of these features. To answer this question, we focused on patients with a diagnosis of type 2 diabetes who had at least one uncontrolled physiological measure (hemoglobin A1c, LDL cholesterol, blood pressure) at baseline (2009-2010) to examine whether those who had used the portal’s Web-based prescription refill or SM features for the first time between 2010 and 2013 were more likely than nonusers to achieve control at follow-up (2013-2014). We also sought to explore both the separate and combined effects of Web-based refill and SM use on physiological measures and whether sustained use was associated with a greater probability of achieving control.

## Methods

### Study Design and Overview

We conducted a 5-year retrospective cohort study of Veterans with type 2 diabetes registered for the MHV portal. Data for these analyses came from the Veteran’s Health Administration’s Corporate Data Warehouse, including administrative data, clinical records for inpatient and outpatient care, and MHV registration and use data. We used International Classification of Diseases, Ninth Revision, Clinical Modification (ICD-9-CM) diagnosis codes (October 1, 2007-March 31, 2009) to determine type 2 diabetes diagnosis and determine patient characteristics at baseline. Data from April 1, 2009 through March 31, 2014 were used to assess MHV use over time. Intermediate physiological measures obtained during clinical care were obtained at baseline and follow-up. In addition, we linked income and educational attainment variables from the US Census Bureau’s 2007- 2011 American Community Survey (5-year estimates) to each Veteran via postal code.

### Cohort Eligibility

**Figure 1:**
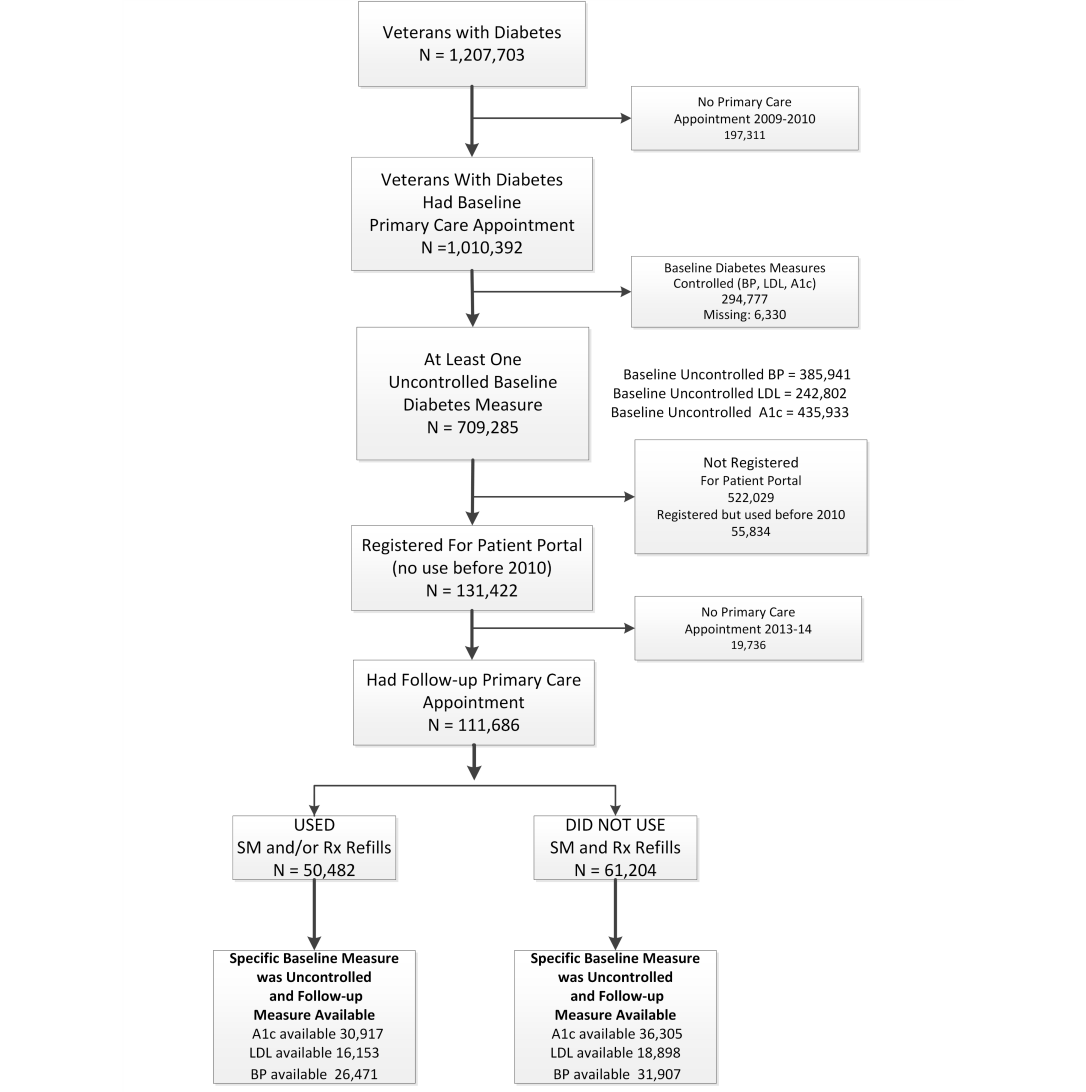
Cohort Selection.

We identified patients who had at least two outpatient records or one inpatient record with an ICD-9-CM diagnosis code for type 2 diabetes by March 2009 (N=1,207,703). Use of two or more diabetes-related ICD-9-CM codes from inpatient or outpatient visits has previously been determined to be the most accurate way to identify patients with diabetes in VA administrative data [24]. We then excluded patients who had not used the VA for primary care in 2009 to 2010, who had controlled or missing diabetes outcome measures, who were not registered for the portal, or who had used the MHV Web-based prescription refill or SM features before 2010 (see Figure 1). We limited our analyses to those who were registered to use the MHV portal to minimize differences in access to the portal or in willingness to use the portal among users and nonusers so that we could focus on associations with actual use. Our previous work has shown patients registered for the portal (but not using features) to be a more appropriate and comparable reference group [7]. Because our goal was to understand how a patient portal could assist in achieving improvements in physiological control, we also excluded those who were controlled at baseline from the main analyses as those patients had already successfully managed to control their physiological measures without the use of MHV. The final analysis cohort included 111,686 patients.

### Variables

#### Dependent Variables—Diabetes-Related Physiological Measures: HbA1c, LDL, Systolic and Diastolic Blood Pressures

We used the American Diabetes Association’s guidelines to define cutoffs for glycemic, cholesterol, and blood pressure control [25]. We defined uncontrolled blood glucose at baseline (April 2009-March 2010) if the patient’s average hemoglobin A1c (HbA1c) during that period was greater than or equal to 7.0% (53 mmol/mol) and uncontrolled LDL cholesterol at baseline if the patient’s average cholesterol reading during that same period was greater than or equal to 100 mg/dL (2.586 mmol/L). Similarly, we determined that a patient had uncontrolled blood pressure if the average systolic blood pressure at baseline (assessed by averaging all readings during the baseline year) was 140 mmHg or higher, or the average diastolic blood pressure (similarly averaged across the baseline year) was 80 mmHg or higher. To achieve control by follow-up, patients had to lower their readings to below the cutoffs (blood glucose and LDL cholesterol) or achieve control over both systolic and diastolic blood pressures (blood pressure). A binary indicator for whether a patient with uncontrolled values at baseline achieved control by follow-up (2013-2014) was the dependent variable for the logistic regression models.

#### Independent Variables—Use of the Portal: Registration, Use of Web-Based Prescription Refill and Secure Messaging

Among Veterans registered by April 1, 2013, we measured use of two key features of the portal, which had been available throughout the study period: the Web-based prescription refill feature and the SM feature and used a binary indicator of any use to describe the samples. We assessed how often each patient used each feature during the potential exposure period (April 2010-March 2013). At some facilities, patients were prompted to try these features (eg, send a test message to one’s primary care team) as part of a MHV training. We therefore defined “use” as two or more prescriptions filled online via the MHV portal per year or two or more SMs sent per year, to ensure we captured actual use and not just attendance at a training session. To measure dose of exposure, our primary measure of use for each portal feature was a categorical variable indicating whether a patient had used each feature two or more times per year over 1 year, 2 years, or for 3 or more years during the potential exposure period. A continuous variable measuring years of use (ie, years with 2+ refills or 2+ SMs sent) for each portal feature was used for tests for trend.

#### Other Covariates

Other covariates we used included demographic characteristics such as patient age, gender, race or ethnicity, urban, suburban, or rural residence, educational attainment, and income. In multivariable models, we adjusted for age, gender, race, comorbidities, and available measures of socioeconomic status because these have been significantly associated with adoption of SM and patient portals in previous studies [9,26]. For income, we included a measure of whether the patient was eligible for free care from the Veterans Health Administration based on low income. Because data on Veterans’ income and educational attainment do not exist in the VA Corporate Data Warehouse, we also linked Census data by postal code of residence on the percentage of adults aged older than 25 years who have attained a bachelor’s degree or higher and the median per capita earnings in the past 12 months (in 2011 inflation-adjusted dollars) among those aged 25 years and older with earnings. We also adjusted for the number of primary care visits a patient had during the baseline year and the number of comorbidities they had as determined by the Elixhauser algorithm for identifying comorbidities from administrative data [27].

### Analyses

We characterized the overall cohort and examined means and distributions of patient demographic and clinical characteristics by use, both overall and for those with specific uncontrolled physiological measures at baseline. We calculated the proportion of patients with diabetes in our cohort using each feature over each year of the study and the average number of prescriptions refilled or secure messages sent during each year. Our primary goal was to assess the association of use of patient portal features with change in diabetes-relevant physiological measures (HbA1c, LDL, BP). To achieve this goal, we first calculated means and binomial confidence intervals (CIs) for the proportion of patients who were uncontrolled at baseline who achieved control at follow-up, stratified by the number of years of use of the SM or Web-based refill features. We then constructed a series of logistic regression models predicting control of each physiological measure at follow-up based on categorical measures of portal use (years of use of each feature), adjusting for the covariates described previously. All logistic regression models were adjusted for patient age; gender; race or ethnicity; eligibility for free VA health care; number of Elixhauser comorbidities at baseline; number of primary care visits at baseline (in 2009-2010) and during the study period (2010-2014); urban, suburban, rural, or highly rural residence; median income by postal code; and the percentage of college graduates in the patient’s residential postal code. In addition, models for control of blood pressure, cholesterol, and HbA1c at follow-up (2013-2014) were adjusted for the patient’s mean baseline blood pressure, LDL cholesterol, or HbA1c value in 2009 to 2010, respectively. Separate models were first run for each feature (Web-based prescription refill use and SM use) because there was a moderate correlation between uses of the two features. To further evaluate the independent effect of each feature, we also ran combined logistic models, which included both Web-based prescription refill use and SM use in the same models. To test for dose response, we then ran tests of trend treating the number of years of use of each feature as a continuous variable. We also conducted sensitivity analyses to see whether results changed depending on (1) our definition of use (ie, defining use as one or more uses of a feature in a given year) or (2) inclusion of patients who met other inclusion criteria but were controlled at baseline in the analysis sample.

## Results

### Feature Use

Within our cohort of 111,686 patients (see Figure 1), 50,482 (45.20%) used Web-based prescription refills or SM or both at least twice per year between April 2010 and March 2014, and 61,204 (54.80%) used neither.

### Patient Characteristics

Table 1 describes the characteristics of the overall sample and examines differences between patients who used the Web-based prescription refill feature or the SM feature or both in MHV between April 2010 and March 2014 and those who did not.

**Table 1:** Characteristics of patients with type 2 diabetes registered for My HealtheVet, overall and by use or nonuse of the Web-based refill or secure messaging features as of March 2014.

Variables		Overall	Used neither Web-based refill nor SM as of March 2014 (nonusers)	Used Web-based refill or SM or both as of March 2014 (users)	Difference between user and nonuser groups (Pearson’s chi-square or 2-sided *t*-test)
**N**		111,686	61,204	50,482	
**Age (mean (SD)**		62.05 (9.6)	63.22 (9.6)	60.63 (9.5)	*t*_111684_ = 45.2, *P*<.001
**Gender (% female)**		3.58%	3.16%	4.08%	χ^2^_1_ = 67.2, *P*<.001
**Race or ethnicity**					
	White	68.87%	67.25%	70.84%	Reference group for χ^2^
	African-American	16.95%	18.64%	14.90%	χ^2^_1_ = 280.3, *P*<.001
	Latino	5.69%	5.63%	5.76%	χ^2^_1_= 1.1, *P*=.294
	Native Hawaiian or Pacific Islander	1.10%	1.08%	1.12%	χ^2^_1_ = 0.1, *P*=.755
	Asian	0.81%	0.73%	0.90%	χ^2^_1_ = 5.9, *P*=.015
	American Indian or Alaska Native	0.71%	0.73%	0.70%	χ^2^_1_ = 1.8, *P*=.178
	Unknown to patient, refused, or missing	5.87%	5.94%	5.79%	χ^2^_1_ = 9.1, *P*=.003
**Percent eligible for free VA health care based on income**		20.43%	21.71%	18.87%	χ^2^_1_ = 137.6, *P*<.001
**Median income in postal code of residence US$ (mean (SD))**		33,548.86 (8,926.98)	33,532.67 (8,996.24)	33,568.51 (8,842.24)	*t*_108985_ = −0.6596, *P*=.5095
**Percent of adults with a university degree or higher in postal code of residence (mean (SD))**		23.46% (12.7 )	23.39% (12.8)	23.54% (12.7)	*t*_109086_= −1.9398, *P*=.0524
**Location**					
	Urban (%)	73.23%	73.27%	73.19%	Reference group for χ^2^
	Suburban (%)	13.40%	13.46%	13.32%	χ^2^_1_ = 0.3, *P*=.597
	Rural (%)	7.23%	7.32%	7.12%	χ^2^_1_ = 1.3, *P*=.259
	Highly rural (%)	6.14%	5.95%	6.38%	χ^2^_1_ = 7.5, *P*=.006
**Number of Elixhauser comorbidities at baseline (mean (SD))**		5.57 (2.5)	5.56 (2.6)	5.59 (2.5)	*t*_111357_=−1.826, *P*=.0679
**Number of primary care visits at baseline (mean(SD))**		4.40 (3.6)	4.37 (3.6)	4.43 (3.6)	*t*_111684_ = −3.0046, *P*=.0027
**Number of primary care visits from 2010 to 2014 (mean (SD))**		17.59 (12.8)	16.97 (12.6)	18.34 (13.0)	*t*_111684_ = −17.86, *P*<.001

Compared with patients who did not use either of the features, patients who used Web-based refill or SM were slightly younger (60.6 years vs 63.2 years, *P*<.001), more likely to be female (4.08% vs 3.16%, *P*<.001), and less likely to be eligible for free VA care based on low economic means (18.87% vs 21.71%, *P*<.001). There were significant differences in race or ethnicity between users and nonusers, with African-American (*P*<.001), Asian (*P*=.015), and patients of unknown race (*P*=.003) less likely than white patients to be users. The difference was most marked between African-American and white patients (39.73% (7,521/18,931) of African-American patients were users vs 46.49% (35,759/76,920) of white patients, *P*<.001). Although most patients resided in urban areas, urban patients were slightly less likely to be users than patients residing in areas designated as highly rural (45.06% (36,078/80,060) vs 46.80% (3,143/6,716), *P*=.006).

There were no significant differences in the number of Elixhauser comorbidities at baseline (*P*=.0679), median income by postal code of residence (*P*=.5095), percentage of adults with a bachelor’s degree or higher in postal code of residence (*P*=.0524). There was a statistically significant difference in the number of primary care visits at baseline in the overall cohort (4.37 visits for nonusers vs 4.43 visits for users at baseline, *P*=.0027), but this difference vanished when looking at analysis subgroups based on uncontrolled measure at baseline (see Table 2). There was a highly significant difference in the number of primary care visits between 2010 and 2014 (16.97 for nonusers vs 18.34 visits for users, *P*<.001). Users also showed evidence of higher primary care utilization in all analysis subgroups (see Table 2).

Further detail describing the characteristics based on each uncontrolled measure (ie, the sample for each logistic regression model) is summarized in Table 2.

**Table 2:** Demographics of patients with type 2 diabetes registered for My HealtheVet by uncontrolled physiological measure at baseline and by use of the portal.

Variables		Uncontrolled Measure					
		Hemoglobin A1c A1c ≥7.0%		Low-density Lipoprotein LDL≥100mg/dL		Blood Pressure BP≥140/80 mmHg	
		Registered, no use	Used SM or Web-based refill	Registered, no use	Used SM or Web-based refill	Registered, no use	Used SM or Web-based refill
**N**		36,305	30,917	18,898	16,153	31,907	26,471
**Age (mean (SD)**		62.66 (9.2)	60.28 (9.2)	61.47 (9.6)	58.68 (9.6)	62.63 (9.7)	60.13 (9.7)
**Gender (% female)**		2.86%	3.71%	4.87%	6.53%	2.88%	3.58%
**Race or ethnicity**						
	White	66.88%	70.98%	64.21%	67.62%	64.55%	68.44%
	African-American	19.12%	14.84%	22.14%	17.56%	21.38%	17.25%
	Latino	6.08%	6.03%	5.79%	6.27%	5.53%	5.63%
	Native Hawaiian Pacific Islander	1.08%	1.11%	1.04%	1.08%	1.08%	1.21%
	Asian	0.75%	0.89%	0.71%	1.00%	0.72%	0.87%
	American Indian or Alaska Native	0.73%	0.70%	0.69%	0.84%	0.71%	0.70%
	Unknown to patient, refused, or missing	5.36%	5.45%	5.42%	5.64%	6.02%	5.91%
**Percent eligible for free VA health care**		22.38%	19.40%	21.61%	18.76%	21.93%	18.96%
**Median income in postal code US$ (mean (SD))**		33,453.54 (8925.47)	33,548.58 (8839.33)	33,111.41 (8813.43)	33,197.85 (8674.04)	33,364.98 (8815.67)	33,424.07 (8763.60)
**Percent adults with a university degree or higher in postal code (mean(SD))**		23.12% (12.7)	23.32% (12.6)	22.95% (12.6)	23.20% (12.4)	23.27% (12.7)	23.46% (12.6)
**Location**							
	Urban (%)	73.21%	73.12%	73.32%	73.43%	73.40%	72.91%
	Suburban (%)	13.34%	13.15%	13.64%	13.15%	13.54%	13.47%
	Rural (%)	7.41%	7.24%	7.18%	7.05%	7.14%	7.12%
	Highly rural (%)	6.04%	6.50%	5.86%	6.37%	5.93%	6.50%
**Number Elixhauser comorbidities at baseline (mean (SD))**		5.70 (2.6)	5.72 (2.5)	5.40 (2.5)	5.45 (2.4)	5.54 (2.5)	5.51 (2.4)
**Number of primary care visits at baseline (2009-10; mean(SD))**		4.64 (3.8)	4.66 (3.7)	4.32 (3.5)	4.38 (3.5)	4.29 (3.5)	4.30 (3.4)
**Number of primary care visits; 2010 to 2014, mean (SD)**		18.04 (12.9)	19.17 (13.3)	17.09 (12.4)	18.29 (12.7)	16.97 (12.5)	18.17 (12.5)

### Portal Use

**Figure 2:**
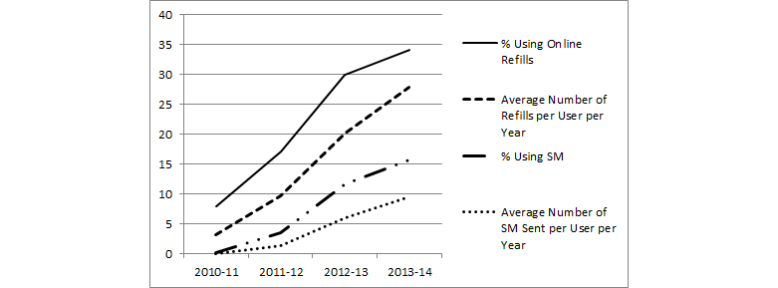
Proportion of patients with type 2 diabetes registered for My HealtheVet and first using Web-based prescription refills or secure messaging after 2010, increase in feature adoption over time, and average number of uses per user per year.

Use of Web-based refills and SM increased steadily from 2010 to 2014 (Figure 2). Among registered patients with diabetes who had not used the portal before 2010, only 7.98% (8,917/111,686) used Web-based prescription refills in 2010 to 2011, and the average number of refills per year was 3.13 per user. In the same year, as SM was just being implemented at most facilities, only 0.22% (241/111,686) used SM and sent an average of 0.059 messages per user. By 2013 to 2014, the numbers had risen to 34.13% (38,113/111,686) of new users using Web-based refills, filling an average of 27.84 prescriptions each, and 15.75% (17,592/111,686) were using SM, sending an average of 9.46 messages each.

### Association of Patient Portal Use and Change in Diabetes Physiological Measures over 5 Years

The logistic regression results are presented in Table 3.

Our single-feature logistic regression models (Models 1a-c and Models 2a-c) showed that patients with uncontrolled HbA1c at baseline (2009-2010) were significantly more likely to achieve glycemic control at follow-up (2013-2014) if they used SM for 2 or more years. The odds of having an HbA1c below 7.0% (53 mmol/mol) at follow-up were 22% higher (after 2 years of use, odds ratio: OR=1.22, CI: 1.13-1.32) and 28% higher (after 3 or more years, OR=1.28, CI: 1.13-1.44), for those using SM compared with those who never used it.

However, use of Web-based prescription refills was only associated with glycemic control at follow-up after 3 or more years of use (OR=1.07, CI: 1.01-1.14). Those with uncontrolled blood pressure at baseline were significantly more likely to achieve control at follow-up only with 2 (OR=1.06, CI: 1.01-1.12) or 3 or more (OR=1.05, CI: 1.00-1.11) years of Web-based refill use, compared with nonusers. Use of SM was not significantly associated with improvements in blood pressure control. Both Web-based refill use and SM use were significantly associated with improvements in LDL cholesterol levels at follow-up. Compared with nonusers, the odds of users having LDL cholesterol below 100 mg/dL (2.586 mmol/L) were 12% higher with 2 years of Web-based refill use (OR=1.12, CI: 1.05-1.20), 16% higher with 3+ years of Web-based refill Use (OR=1.16, CI: 1.08-1.24), 9% higher with 1 year of SM use (OR=1.09, CI: 1.01-1.18), 17% higher with 2 years of SM use (OR=1.17, CI: 1.07-1.27), and 22% higher with 3+ years of SM use (OR=1.22, CI: 1.06-1.40).

We also ran logistic regression models identical to those mentioned previously that included both years of SM and Web-based refill use in the same model (Models 3a-c), as well as logistic regression models that included years of SM or Web-based refill use as a continuous variable as a test for trend (Models 4a-c). The conclusions remained largely unchanged, although ORs for the association between SM use and LDL were more attenuated (and no longer significant with the exception of 2 years of SM use) in the combined model. The combined model (and test for trend) did not show a significant association between SM use and blood pressure control (*P*=.370 for trend), or between Web-based refill use and glycemic control (*P*=.585 for trend); however, tests for trend revealed significant dose-response relationships between use of SM and glycemic control (*P*<.001), use of Web-based refill and blood pressure control (*P*=.001), and use of both features and LDL control (*P*<.001 and *P*=.015 for trend, respectively, for refills and SM use). 

**Table 3:** Adjusted odds of being in control at follow-up (OR (95% CI)) for a patient with uncontrolled physiological measures (HbA1c, LDL, or blood pressure) at baseline, based on years of portal feature use.

Models^a^		Health Outcomes in 2013-14		
		**Hemoglobin A1c** HbA1c<7% (53 mmol/mol)	**Low-density Lipoprotein** LDL < 100 mg/dL (2.586 mmol/l)	**Blood Pressure** SBP<140 mmHg DBP<80 mmHg
**Models 1a-c: adjusted odds ratios (95% CI) for being controlled in 2013-2014 among patients with uncontrolled physiological measures in 2009-10 based on years of Web-based prescription refill use^a^**				
Web-based prescription refill use				
	None	Reference	Reference	Reference
	1 year	0.99 (0.93, 1.05)	1.01 (0.95, 1.08)	1.02 (0.97, 1.08)
	2 years	1.01 (0.95, 1.08)	1.12 (1.05, 1.20)^c^	1.06 (1.01, 1.12)^b^
	3 or more years	1.07 (1.01, 1.14)^b^	1.16 (1.08, 1.24)^d^	1.05 (1.00, 1.11)^b^
**Models 2a-c: adjusted odds ratios (95% CI) for being controlled in 2013-2014 among patients with uncontrolled physiological measures in 2009-2010 based on years of secure messaging use^a^**				
Secure messaging use				
	None	Reference	Reference	Reference
	1 year	1.03 (0.96, 1.10)	1.09 (1.01, 1.18)^b^	1.03 (0.97, 1.09)
	2 years	1.22 (1.13, 1.32)^d^	1.17 (1.07, 1.27)^c^	1.03 (0.96, 1.10)
	3 or more years	1.28 (1.13, 1.44)^d^	1.22 (1.06, 1.40)^c^	1.00 (0.90, 1.12)
**Models 3a-c: adjusted odds ratios (95% CI) for being controlled in 2013-2014 among patients with uncontrolled physiological measures in 2009-2010 based on years of both features^a^**				
Web-based prescription refill use				
	None	Reference	Reference	Reference
	1 year	0.96 (0.91, 1.03)	1.01 (0.94, 1.08)	1.02 (0.97, 1.07)
	2 years	0.96 (0.90, 1.03)	1.13 (1.05, 1.21)^c^	1.07 (1.01, 1.13)^b^
	3 or more years	1.00 (0.94, 1.07)	1.13 (1.05, 1.22)^c^	1.08 (1.02, 1.14)^c^
Secure messaging use				
	None	Reference	Reference	Reference
	1 year	1.04 (0.97, 1.12)	1.05 (0.97, 1.14)	1.00 (0.94, 1.07)
	2 years	1.24 (1.14, 1.34)^d^	1.10 (1.00, 1.21)^b^	0.98 (0.91, 1.05)
	3 or more years	1.28 (1.12, 1.45)^d^	1.12 (0.96, 1.30)	0.95 (0.85, 1.07)
**Models 4a-c: combined tests for trend predicting controlled outcomes in 2013-2014 among patients with uncontrolled physiological measures in 2009-2010 based on years of use for both features^a^**				
Web-based prescription refill use		*P*=.585	*P*<.001	*P*=.001
Secure messaging use		*P*<.001	*P*=.015	*P*=.370

^a^All models adjust for patient characteristics in Table 1 including age, gender, race or ethnicity, eligibility for free care, geographic location, number of Elixhauser comorbidities, and baseline number of primary care visits in 2009 to 2010. In addition, models adjusted for the patient’s physiological measure (blood pressure, LDL cholesterol, or HbA1c value) in 2009 to 2010, median income in the patient’s residential zip code, and the percentage of college graduates in the patient’s residential postal code.

^b^Odds ratios are significant at the *P*<.05 level as indicated.

^c^Odds ratios are significant at the *P*<.01 level as indicated.

^d^Odds ratios are significant at the *P*<.001 level as indicated.

**Figure 3:**
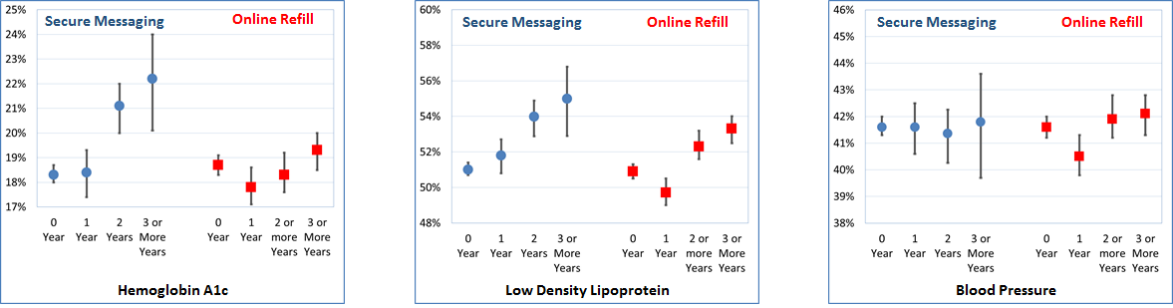
Proportion controlled at follow-up, out of all diabetics uncontrolled for that specific measure at baseline (proportion and binomial CIs).

Figure 3 shows the predicted probability of achieving control over each measure by follow-up based on years of refill and SM use among those uncontrolled at baseline for each measure. The figure illustrates how sustained use of each tool is associated with improvements in control of physiological measures.

### Sensitivity Analyses

We conducted sensitivity analyses to see whether our results would change with the inclusion of those whose physiological measures were controlled at baseline, but otherwise met criteria for inclusion. Although the ORs were attenuated, significant tests for trend revealed the same relationships between feature use and being in control at follow-up for all the measures. Similarly, when use was defined as use of a feature even once in a given year, ORs were again somewhat attenuated; however, the results, including the tests for trend, led to identical conclusions about the associations between feature use and controlled physiological outcomes at follow-up.

## Discussion

### Principal Findings

Within this cohort of patients with type 2 diabetes and uncontrolled physiological measures, we saw increasing activity on the MHV patient portal between 2010 and 2014. The rate of use and increase in use was greater for Web-based refills than for SM. We observed small, statistically significant, and potentially meaningful improvement in physiological measures among diabetic patients who initiated and sustained use of Web-based refills or SM or both via MHV. However, the association varied by specific MHV feature. Where a significant association was found, use of SM was associated with higher odds of improved outcomes than use of Web-based refills.

### Comparison With Prior Work

The association between use of SM and improved diabetes physiological measures is consistent with that of prior research [14-16]; however, we were able to add information on the effects of sustained use over many years. For most measures, we found a dose-response effect on outcomes, suggesting that sustained use of the feature was associated with greater likelihood of being controlled at follow-up. The more years the patient used the feature, the greater the odds of achieving control compared with those who did not use the feature. Use of SM was associated with improvements in glycemic control with sustained use over 2 to 3+ years. Type 2 diabetic patients with uncontrolled blood pressure were more likely to achieve blood pressure control with 2 to 3+ years’ use of Web-based medication refills through MHV. Both prescription refills and SM were associated with improvements in lipid levels with sustained use. Adjusting for use of both the features in the model did shift the magnitude of the odds of achieving control. This suggests that the association between patient portal use and health outcomes will vary based on the combination of different features used and how patients are using each feature for self-management of their health conditions.

One mechanism by which Web-based medication refills may affect health outcomes may be through improved adherence to prescribed medications. In prior work, MHV use has been associated with improvements in antiretroviral adherence [28]. To the extent that Web-based refills increase the likelihood of refilling prescriptions, they may improve availability of medications, which may lead to improvements in adherence. If the Web-based refill feature improves adherence to antihypertensives and statins, they are likely to improve hypertension and lipid control over time. However, we did not see an association between sustained use of Web-based refills and improvements in HbA1c levels. Because HbA1c is a measure of blood sugar levels over several months, it may take a longer time for improved adherence to diabetes medications to result in measurable improvements, unlike blood pressure and LDL cholesterol, which can result in more rapid improvements even with improved short-term adherence to antihypertensives and statins. A patient’s blood sugar levels are also more sensitive to patient diet and self-management, as well as adequate medication titration, both of which may require more patient–provider communication and clinician support to achieve.

SM has been shown to improve patient ratings of patient–provider communication [29]. Thus, SM may also affect adherence by facilitating patient–provider communication about medication or behavioral concerns, which are barriers to adherence. It may also facilitate coordination of care and make it easier for primary care clinicians to refer patients to other related services such as nutrition consults, diabetes counseling, or weight loss programs [30]. This may explain why glycemic control, which requires significant and often complex patient self-management in addition to medication management, was found to be significantly associated with sustained use of SM.

This work also expands on previous research that has often focused generally on the patient portal or PHR use [12] or the use of a particular feature such as SM [9,14,17], without accounting for their relative effects when used in combination with other features. As features continue to be added to portals, further research should continue to examine the effects of different portal features both separately and in combination, to determine which features are most effective at improving the specific patient outcomes of interest.

Patients who used one or both features during the study period were more likely to be younger, female, white, and were less likely to be socioeconomically disadvantaged than other patients with diabetes who met our inclusion criteria. Numerous studies have documented sociodemographic differences in patient portal access and adoption [26,31-33]. Although we attempted to minimize differences in access by limiting our analyses to patients who had registered for the portal, we still observed differences across groups. It is important to ensure that any improvements in health status achieved through the patient portal do not further widen existing disparities in health because of disparities in portal access or adoption. Lyles et al found that racial or ethnic difference in diabetic patients’ shared medical record use was not fully explained by differences in patient sociodemographics, patient health status, or provider encouragement of SM [26]. We will have to be mindful of these potential disparities and specifically target vulnerable patients with support interventions for use of portal features found to significantly affect health outcomes.

### Limitations

There are a number of limitations to this study. The VA patient portal has been deployed nationwide. As all patients are free to choose whether to use the patient portal, it is difficult to limit access or to randomize access to various features to conduct a randomized controlled trial. Because this is an observational study, it is impossible to ensure that the comparison group (ie, the nonusers) is similar in all ways to the portal users. As discussed, we limited the sample to those who had registered to use the portal to reduce heterogeneity in measured and unmeasured confounders. In our prior research [7], we have demonstrated that demographic characteristics were more similar when comparing registered users and nonusers, versus comparing those registered and those not registered. By using patients with diabetes who had registered for MHV (but not used the prescription refill or SM features more than once, if ever,) as a comparison group, we minimized some of this bias by limiting our analyses to patients who had access to the portal and who had attended a training or otherwise shown an interest in using it at some point. We saw that the patients in the comparison group for each logistic regression model were very similar in terms of their baseline health care utilization (number of primary care visits) and number of Elixhauser comorbidities (see Table 2). However, without a measure of patient engagement, there is still the possibility that patients may self-select to use these features precisely because they are already more engaged in their care; the lack of a measure of patient engagement is another limitation of this study. Randomized encouragement trials [34] may be one method to strengthen the rigor of future work.

### Conclusions

Recognizing that our study is an observational study and that the associations cannot be considered causal, the availability of multiple years of observational data, detection of a dose response, and adjustment for patient characteristics known to influence technology use and diabetes outcomes strengthen the potential conclusions we can draw from this analysis about the differential effects use of patient portal features may have on physiological outcomes. The results in this study suggest that measuring the relative use and relative association of each feature of a patient portal is critical because each can have a different effect on changes in health care and health outcomes.

Future research should also focus on uncovering the mechanisms (causal pathways) through which portal use leads to physiological improvements. Does improved communication with providers via SM lead to greater patient engagement between visits, sustained behavior changes, better continuity of care, improved medication titration by the clinical team, or improved adherence to medications by the patients? What portion of the engagement might be explained by other portal features such as the ability to track and chart their blood glucose or blood pressure measurements? A study of adult diabetes patients at Kaiser Permanente found that both patient nonadherence to medications for glycemic, lipid, or blood pressure control and lack of provider treatment intensification occurred frequently among patients whose outcomes are above desired target levels [35]. It may be that portal use assists with patient adherence to medications by facilitating prescription refills, and that patient–provider communication between face-to-face visits can lead to improvements in levels of appropriate treatment intensification by providers. These pathways must be better understood to leverage portal features for interventions.

## Abbreviations

BP: blood pressure
HbA1c: hemoglobin A1c
ICD-9-CM: International Classification of Diseases, Ninth Revision, Clinical Modification
LDL: low density lipoprotein
MHV: My HealtheVet
PHR: personal health record
SM: secure messaging
VA: Department of Veterans Affairs
